# Individual variability in space use near power lines by a long‐lived territorial raptor

**DOI:** 10.1002/ece3.8811

**Published:** 2022-04-07

**Authors:** Ana Teresa Marques, Luís Palma, Rui Lourenço, Rogério Cangarato, Alexandre Leitão, Miguel Mascarenhas, João Tiago Tavares, Ricardo Tomé, Francisco Moreira, Pedro Beja

**Affiliations:** ^1^ CIBIO Centro de Investigação em Biodiversidade e Recursos Genéticos InBIO Laboratório Associado Universidade do Porto Vairão Portugal; ^2^ CIBIO Centro de Investigação em Biodiversidade e Recursos Genéticos InBIO Laboratório Associado Instituto Superior de Agronomia Universidade de Lisboa Lisboa Portugal; ^3^ BIOPOLIS Program in Genomics, Biodiversity and Land Planning CIBIO Vairão Portugal; ^4^ MED Instituto Mediterrâneo para a Agricultura Ambiente e Desenvolvimento, CHANGE Laboratório Associado LabOr – Laboratório de Ornitologia IIFA Universidade de Évora Évora Portugal; ^5^ 360 Graus ‐ Cultura e Ambiente Évora Portugal; ^6^ Strix Parede Portugal; ^7^ Bioinsight Odivelas Portugal; ^8^ R. Francisco Xavier Guedelha Faro Portugal

**Keywords:** *Aquila fasciata*, attraction, avoidance, individual behavior, infrastructures, power lines

## Abstract

Evaluating species responses to anthropogenic infrastructures and other habitat changes is often used to assess environmental impacts and to guide conservation actions. However, such studies are generally carried out at the population level, disregarding inter‐individual variability. Here, we investigate population‐ and individual‐level responses toward power lines of a territorial raptor, the Bonelli's eagle A*quila fasciata*. We used GPS‐PTT tracking data of 17 adult eagles to model space use as a function of distance to transmission and distribution lines, while accounting for other habitat features known to affect this species. At population level, eagles increased the intensity of space use in the proximity of power lines (up to 1,000 m), suggesting an attraction effect. At individual level, some eagles shared the general population attraction pattern, while others showed reduced intensity of space use in the proximity of power lines. These differential responses were unrelated to the sex of individuals, but were affected by the characteristics of the power grid, with a tendency for apparent attraction to be associated with individuals occupying home ranges with a denser network of transmission lines and transmission pylons. However, the study could not rule out the operation of other potentially influential factors, such as individual idiosyncrasies, the spatial distribution of prey availability, and the availability of natural perches and nesting sites. Overall, these results suggest that power lines may drive different behaviors and have differential impacts across individuals, with those attracted to the proximity of power lines potentially facing increased risk of mortality through electrocution and collision, and those avoiding power lines being potentially subject to exclusion effects. More generally, our results reinforce the need to understand individual variability when assessing and mitigating impacts of anthropogenic infrastructures.

## Graphical abstract

We found power lines may drive different behaviors and have differential impacts across individuals. Our results reinforce the need to understand individual variability when assessing and mitigating impacts of anthropogenic infrastructures.

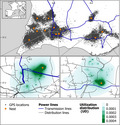

## 1 INTRODUCTION

Understanding animal responses to habitat features is critical for species conservation, as it underpins management strategies to promote long‐term population viability (Fryxell et al., 2014). However, there is increasing evidence that habitat selection patterns may differ across individuals from the same population (Bonnot et al., 2015; Campioni et al., 2012; Leclerc et al., 2016; Lesmerises & St‐Laurent, 2017; Ofstad et al., 2019). This can be due to intrinsic variation among individuals, with genetic or developmental history resulting in distinct personalities; or it can be driven by external factors or internal state (Hertel et al., 2020). Whatever the reasons, variability in habitat preferences may have consequences on individual survival and thus on population processes, particularly when animals face risk‐related trade‐offs, with, for instance, certain individuals preferring habitats with higher mortality risk but abundant foraging resources, while others prioritize safer habitats despite having less resources (Ciuti et al., 2012; Haus et al., 2020). These observations suggest that, at least in some cases, conservation management should be adjusted to meet the distinct requirements of multiple individuals (Merrick & Koprowski, 2017), although the circumstances under which this may be necessary remain poorly known.

Reducing the impacts of a growing network of anthropogenic infrastructures (e.g., roads, railways, and power lines) is becoming a conservation priority worldwide, requiring a better understanding of intra‐population variation in the behavioral responses to these novel habitat features. A wealth of studies has shown that these structures can greatly affect animal behavior in a number of ways causing, for instance, changes in home ranges, movement patterns, reproductive success, escape responses, and physiological states (Coffin, 2007; Trombulak & Frissell, 2000). Changes in space use and movement patterns are probably the most noticeable behavioral effects, which occur because individuals (i) avoid the infrastructure itself, (ii) avoid the disturbance or risks associated with the structure (e.g., traffic, noise, lights, pollution, and incoming predators), or conversely (iii) are attracted to it (Rytwinski & Fahrig, 2015; Walters et al., 2014). Behavioral patterns may even change across the life of an individual, particularly in long‐lived species, due to habituation or learning processes. For instance, white‐tailed eagles *Haliaeetus albicilla* have high mortality levels due to collision with wind turbines, but while adult birds avoid wind farms, the same does not happen in subadults (Dahl et al., 2013). Differences in individual responses toward infrastructures can also result from other factors, depending, for instance, on the particular habitat context, life experiences, and intrinsic processes. So far, however, there is limited understanding on individual behavior towards infrastructures, although such information can play an important role in planning and mitigating their impacts.

Overhead power lines are ubiquitous across vast areas, having the potential to trigger differential behavioral reactions in individuals of the same population. Besides crossing these structures during flight, birds may use pylons as hunting and roosting perches, as well as nesting platforms (Biasotto & Kindel, 2018; D’Amico et al., 2018). Such interactions may cause mortality due to electrocution or collision, or otherwise have positive effects by providing safe nesting sites or enhancing predation efficiency in raptors (Bernardino et al., 2018; Biasotto & Kindel, 2018; D’Amico et al., 2018). Additionally, power lines are tall artificial structures that may change habitat use by causing avoidance behaviors, which may result in habitat loss and fragmentation. Displacement caused by tall infrastructures has been particularly described in birds from open landscapes, and are associated to increases in perceived predation risk, as predators like raptors often use pylons as vantage points, or to neophobia, in reaction to extraneous artificial features (Biasotto & Kindel, 2018; Walters et al., 2014). Therefore, attraction or avoidance toward power lines may have consequences on individual fitness and survival, and ultimately may result in population effects.

In this study, we aimed at evaluating individual responses toward power lines, using the Bonelli's eagle A*quila fasciata* as the model species. This is a long‐lived, resident and territorial raptor, currently categorized as Near Threatened in Europe (BirdLife International, 2015). The species is highly affected by mortality due to electrocution on electric pylons, while collisions with overhead wires have been reported only occasionally (Hernández‐Matías et al., 2015; Real et al., 2001; Rollan et al., 2010). To our best knowledge, behavioral avoidance of power lines has not been reported in this species, but such potential effect has raised concerns from conservation agencies during the licensing processes of new power lines, sometimes resulting in requests for companies to implement mitigation or compensatory measures. The study was conducted in southern Portugal, focusing on an eagle population that has been regularly monitored in the last 25 years (Dias et al., 2017; Palma et al., 2013). Since 2006, eagles in this population have been tracked with GPS PTT tags, providing the opportunity to evaluate interactions with both transmission and distribution lines located within their home ranges. Using this dataset, we analyzed habitat selection by Bonelli's eagles in relation to the spatial distribution of power lines and other habitat features, thereby assessing the extent to which the power line network affects space use by these eagles at (i) population and (ii) individual levels, and (iii) exploring the potential drivers of variability in individual responses to power lines. Results were then used to discuss how variations in behavioral responses across individuals should be accounted for when evaluating and mitigating the impacts of power lines.

## 2 MATERIAL AND METHODS

### 2.1 Study area

**FIGURE 1:**
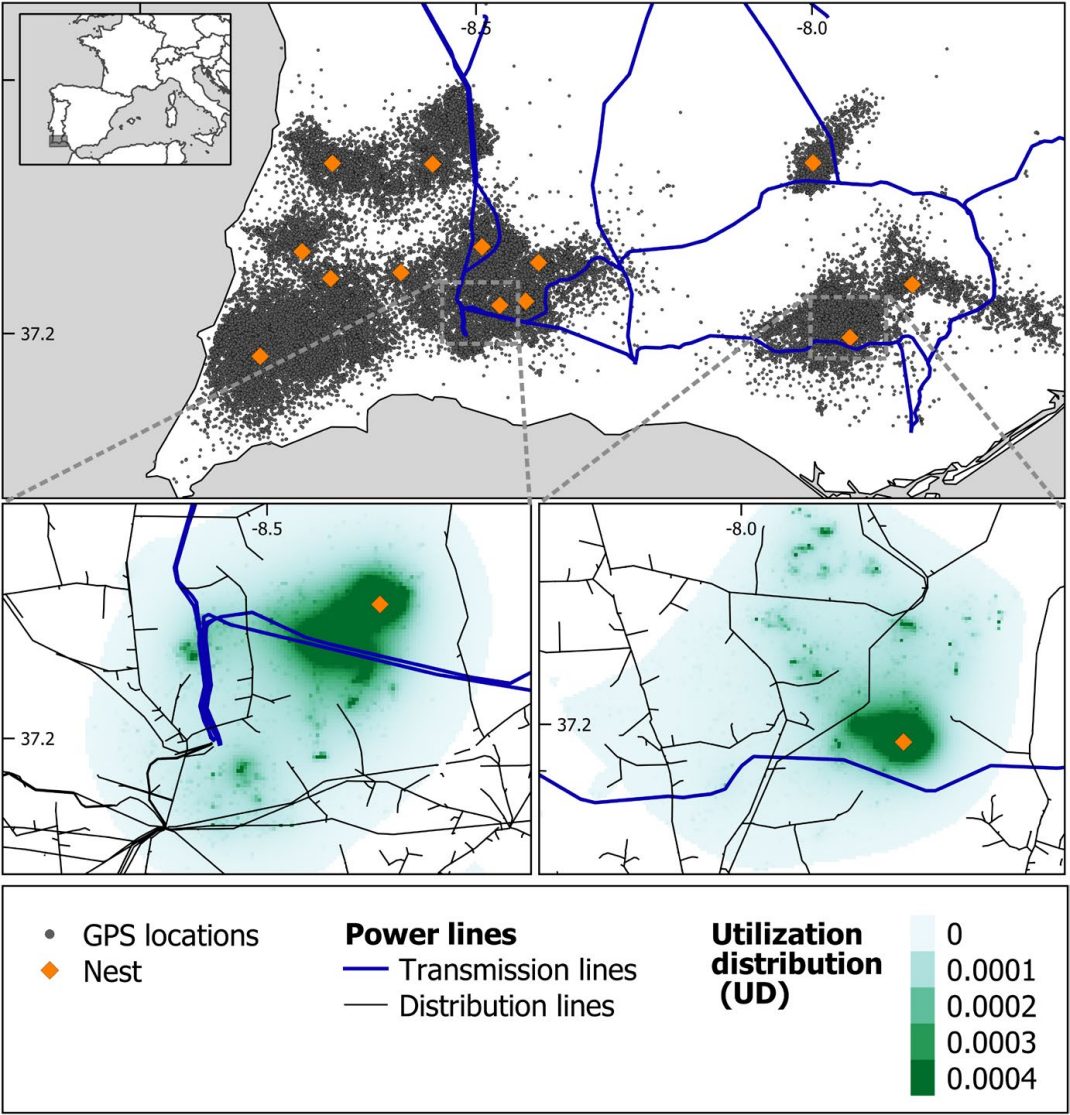
Location of the study area in Southern Portugal, showing the spatial distribution of nests and GPS tracking data of Bonelli's eagles tracked in the study area (top panel). Lower panels show examples of use intensity maps for eagles with increased (bottom left panel) or reduced space use (bottom right panel) in the proximity of power lines

This study was carried out in the uplands of the Algarve (<902 m a.s.l.) and the southern Alentejo peneplain, in the south of Portugal (Figure 1). Land cover consists of open to dense cork oak *Quercus suber* woodlands, with extensive understory scrub dominated by gum cistus *Cistus ladanifer*, and areas with blue gum *Eucalyptus globulus* plantations and scattered small pine *Pinus* spp. stands. The area has been affected by severe and repeated wildfires in the past decades, thereby increasing land cover by scrublands (Acácio et al., 2009). Within eagles’ home ranges, human population density is low and the road network is sparse, and the electricity grid includes distribution (mainly 15, 30, and 60 kV) and transmission power lines (150 and 400 kV). Following management prescriptions issued by the Portuguese conservation authority, new infrastructures must adopt pylon designs that prevent raptor electrocutions and power line routing should avoid the proximity of Bonelli's eagles nests (ICNF, 2019). The Bonelli's eagle population from southern Portugal is almost exclusively tree nesting (Palma et al., 2013) and is genetically divergent from neighboring populations (Mira et al., 2013), which are mainly cliff nesting (Hernández‐Matías et al., 2013). Population size has been increasing at least since the early 1990s, from ca. 33 breeding pairs in 1991 to ca. 100 pairs in 2013 (Palma et al., 2013), and is still growing (L. Palma, unpublished data). While there is no published information on responses to power lines in this population, data from southern Portugal suggest that electrocutions affect mainly juveniles and immature birds (Sousa, 2017), while collisions with power lines have not been recorded.

### 2.2 Study design

The study was designed to model how the use of space by Bonelli's eagles is affected by the proximity to power lines, while controlling for other potentially influential factors. Significant increases or decreases in space use near power lines were taken to indicate attraction or avoidance behaviors, respectively, in common with the assumptions generally adopted in habitat selection studies (Capra et al., 2017; Haus et al., 2020; Mercker et al., 2021). Yet, we also assumed that such effects may be a consequence of responses either to the power lines themselves, or to unmeasured habitat conditions that resulted from their installation, such as changes in vegetation or prey availability under and around lines and pylons (Dupras et al., 2016; Ferrer et al., 2020). Modeling was based on the resource utilization function (RUF) framework (Marzluff et al., 2004), using a dataset of diurnal PTT GPS locations obtained at 1‐h intervals, from 17 adult eagles tracked between 2006 and 2020. The framework involves a two‐step analysis, first estimating the intensity of space use (i.e., utilization distribution [UD]) of each individual within its home range, and then linking the space use to a set of spatially explicit covariates in a regression model (Hooten et al., 2017). The dataset used in analysis was truncated to a maximum distance of 2,000 m from power lines, assuming that the strongest behavioral responses should occur at relatively short distances from lines, and to reduce potentially confounding effects of other factors affecting space use farther away from lines. Modeling was first carried out using data from all individuals, thereby estimating the mean responses of the population to the predictors, and then models were estimated separately for each eagle, thereby evaluating individual‐specific responses. Finally, we used the individual response curves to estimate whether each eagle showed avoidance or attraction behavior to the proximity of power lines, and we built a regression model to estimate whether such contrasting responses were affected by home range characteristics.

### 2.3 Bird data

Seventeen adult eagles (10 females and 7 males) from 13 breeding territories were fitted with Microwave Telemetry Inc. (Columbia, MD, USA), solar GPS PTT‐100 satellite transmitters (Table S1). Eagles were captured in their territory outside the breeding season with a baited trap. Tracking devices were attached as backpacks using a Teflon harness, overall weighing less than 3% of the birds’ mass, and programmed to collect location data at 1‐h intervals during daylight. These settings were selected to focus on eagles’ displacements when activity is expected (i.e., during the day), while maximizing battery life. Although one non‐territorial individual was also tracked, it was discarded because its movement patterns differed greatly from those of territorial individuals (Balbontín & Ferrer, 2009; Cadahía et al., 2010). Likewise, we discarded data from the dispersal phase and included just the territorial period for three other individuals. Eagle trapping and GPS tagging were carried out under license from the Portuguese national authority for nature conservation (ICNF; permits 317/2008, 318/2008, 319/2008, 229/2009, 230/2009, 32/2011, 33/2011, 34/2011, 394/2012, 395/2012, and 396/2012), following approved procedures to maximize animal welfare and reduce risks to the eagles.

### 2.4 Habitat variables and infrastructure data

In modeling, we considered a predictor related to the power line network, plus five additional predictors reflecting land cover, topography, intra‐specific interactions, and road networks (Table S2), which were also expected to influence habitat selection by Bonelli's eagles (Di Vittorio et al., 2012; Dias et al., 2017; Muñoz & Real, 2013; Real et al., 2016). All predictors were extracted to a raster grid with 100 m resolution, using the “raster” package (Hijmans & Van Etten, 2021) in R software (R Core Team, 2020). Land cover data were extracted from Portugal's 2007 Land Cover Map (DGT, 2007), and it was aggregated in five broad categories reflecting habitats potentially influencing Bonelli's eagles in the region (Dias et al., 2017; Palma et al., 2006). Terrain ruggedness was calculated as the mean of absolute differences between the elevation of a cell and that of the surrounding cells (Wilson et al., 2007), using data from the ASTER Global Digital Elevation Model with 30 m resolution (NASA JPL, 2009). Because space use can be constrained by nest site location, we estimated the distance of each raster cell to the main nest (most often used) within each eagle home range. Seemingly, as eagles likely avoid the centers of activity of other breeding pairs, we also estimated the distance of each raster cell to the nearest main nest of neighboring pairs. We only considered conspecifics, as potential competitors, as other large raptors occurred only sporadically in our study area, with the exception of short‐toed eagles (*Circaetus gallicus*), which were common but very rarely showed agonistic interactions with Bonelli's eagles (L. Palma, unpublished data). We mapped the electric grid within the eagles’ home ranges using information provided by REN – Redes Energéticas Nacionais and EDP – Energias de Portugal. We distinguished between transmission and distribution power lines, as the former are taller and have higher pylons that can provide nesting locations or vantage points for hunting, but they may also displace individuals avoiding large anthropogenic structures (APLIC, 2012), while distribution lines tend to be smaller and more unobtrusive, but they can represent an electrocution risk, depending on pylon design (Slater et al., 2020). Because transmission lines were absent from many territories, we used one variable considering the distance of each raster cell to the nearest power line irrespective of typology, and another considering the nearest distance to distribution lines. Differential responses to these variables were expected to indicate an effect of transmission lines. Finally, we estimated the distance to the nearest paved road based on OpenStreetMap (Haklay & Weber, 2008).

To model factors correlating with increased use (attraction) versus decreased use (avoidance) of space in the proximity to power lines, we estimated within each eagle home range: (i) minimum distance between the nest location and power lines (m); (ii) power line density (km/km^2^); and (iii) total number of pylons per home range. Each metric was computed considering both all power lines, and separately the transmission and distribution lines.

### 2.5 Habitat selection modeling

We used Brownian bridge movement models (BBMMs; Kranstauber et al., 2012) to estimate the diurnal utilization distribution (UD) of each eagle for our study area grid. These models include the distance and elapsed time between successive fixes, as well as the GPS location error and the Brownian motion variance, calculating the UD based on the movement path of animals rather than individual locations (Horne et al., 2007; Kranstauber et al., 2012). BBMMs were calculated per eagle, producing a raster layer where the sum of all cells is 1, thus making comparisons across individuals independent of sampling effort. We computed a global BBMM model per eagle, including all GPS fixes, and also separate models for the breeding (January 15th to June 15th) and non‐breeding (June 16th to January 14th) seasons. However, BBMM model results per eagle were highly correlated between seasons (Spearman's rho: Mean ± SD = 0.87 ± 0.08; range = 0.63–0.95), and so we only retained global models in subsequent analysis. Analysis was restricted to eagle home ranges delimited with the 95% UD and to distances up to 2 km from power lines.

To assess the drivers of space use, we used generalized additive mixed models (GAMMs) at the population level and generalized additive models (GAMs) at individual level, both with a Gaussian distribution and an identity link function (Wood, 2017; Zuur et al., 2009). In GAMMs, bird identity was included as a random factor to address dependencies in the replicated measures for each individual (Zuur et al., 2009). There were no problems of multicollinearity among predictors, as their pairwise correlations (all |*r*| < .57) and variance inflation factors (all <3.7) were relatively low (Zuur et al., 2009). We log‐transformed our response variable (bird UD) to obtain a more symmetric distribution and to avoid the overly influence of a few large values. The optimal smoothing parameter was estimated by restricted maximum likelihood (REML), and a basis dimension (*k* = 5) was defined to allow some complexity in the response curves, while avoiding overfitting. Model adequacy was evaluated by plotting residuals versus fitted values and explanatory variables (Zuur et al., 2009).

We visually examined the response curves inferred from individual‐level GAMs, and categorized individuals based on whether there was a general trend for UD consistently declining (attraction) or increasing (avoidance) with distance to lines, within at least the first 1,000 m around lines. Small inflexions of the response curves within this distance range were neglected, as they might reflect local overfitting. We then used univariate generalized linear models (GLM) with binomial distribution and logit link to model attraction (1) or avoidance (0) in relation to the variables describing the power line network within home ranges. We used univariate models because all the predictors were related with the transmission and distribution grids, and therefore highly correlated, and because low sample sizes precluded multivariate modeling. Given the small sample size, we used a *p*‐level of .10 to reduce Type II errors (i.e., rejecting a true effect) (Betensky, 2019). We also tested if the behavior toward power lines differed according to the eagle sex, using a *Q*‐test (Agresti, 2007).

Modeling was done in R (R Core Team, 2020), using the move, mgcv, and stats packages (Kranstauber & Smolla, 2017; Wood, 2018).

## 3 RESULTS

### 3.1 General movement and space use patterns

In total, we tracked the 17 Bonelli's eagles for 18,096 days (mean ± SD: 1,065 ± 754 days/bird; range: 467–3,826), generating 161,973 GPS locations (Table S1, Figure 1). These eagles moved within home ranges of 141.6 ± 71.1 km^2^ (range: 53.4–388.3) km^2^, with no significant differences (*t*‐test: *t* = −1.7303, df = 6.6532, *p* = .1294) between males (181.2 ± 92.8 Km^2^, range: 75.5–388.3) and females (113.9 ± 26.4 km^2^, range: 53.4–154.7) (Table S1). The UD revealed that eagles concentrated their activity in relatively small core areas (UD 50%: 15.6 ± 10.5 km^2^, range: 4.6–43.7), with significantly larger core areas (*t* = −2.941, df = 8.901, *p* = .017) in males (23.6 ± 10.2 km^2^, range: 9.1–43.7) than females (10.0 ± 6.1 km^2^, range: 4.6–21.6) (Table S1).

### 3.2 Population‐level modeling of space use

**FIGURE 2:**
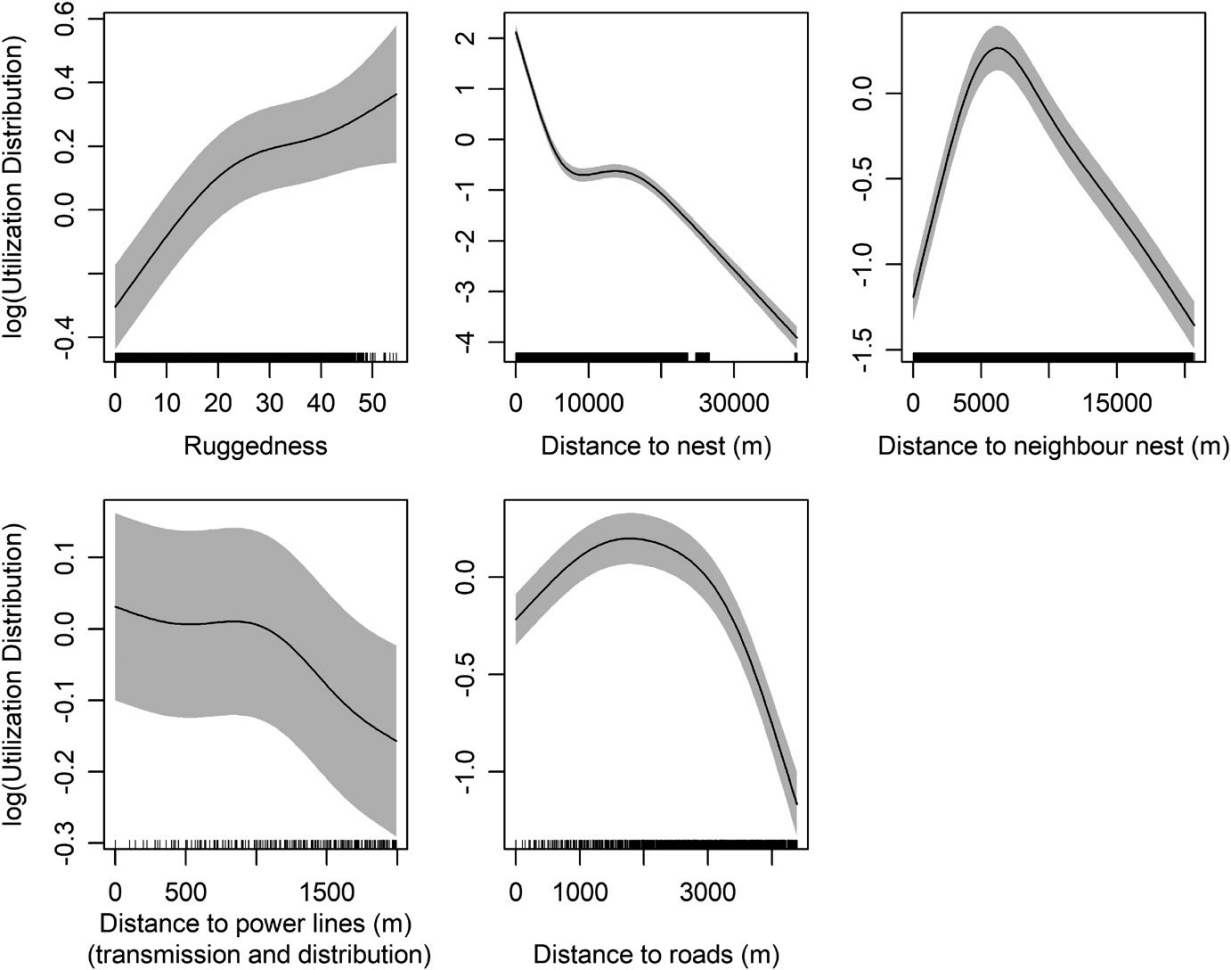
Population‐level partial response curves of Bonelli's eagles inferred from a generalized additive mixed model relating the utilization distribution to predictor variables. Distances to power lines considered both the transmission and distribution network. Shaded areas represent 95% confidence intervals. Ticks on the X‐axis represent the location of observations along the predictor

The overall GAMMs showed that a considerable amount of variation in the intensity of space use by Bonelli's eagles was explained by the spatial distribution of human infrastructures, land cover, topography, and intra‐specific interactions (*R* ^2^ adjusted = 0.474; Table 1, Figure 2, Figure S1). Space use intensity was significantly and inversely related to distance to power lines, showing higher values and little variation within about 1 km of power lines, and then declining farther away. Still, confidence intervals were wide, indicating high uncertainty in the estimation of the mean response curve, and they were much wider than those estimated for the response curves of the other covariates (Figure 2). Such pattern was very similar for both the overall power line network (Figure 2) and the distribution lines (Figure S2). Regarding the other significant covariates, the intensity of space use was low close to roads, increasing up to distances of about 1.5 km, and declining again at larger distances (Figure 2). Space use intensity was also positively related to terrain ruggedness, and it was higher in forests compared to scrubland and, in particular, to agricultural and artificial habitats. Waterbodies were the only land cover category with intensity of use higher than forests (Table 1). Finally, intensity of use declined monotonically with distance to an eagle's own nest, while it increased with distance to the nest of the nearest neighbor up to about 6,000 m, declining farther away (Table 1, Figure 2).

**TABLE 1:** Summary statistics for the generalized additive mixed model (GAMM) for Bonelli's eagle utilization distribution

Model coefficients	Estimate	SE	*t*	edf	*F*	*p*‐value
Intercept	−10.317	0.066	−157.333			<.001
Habitat class (Forest as reference class)						
Artificial	−0.173	0.018	−9.676			<.001
Agriculture	−0.124	0.006	−19.619			<.001
Scrublands	−0.019	0.004	−4.598			<.001
Waterbodies	0.059	0.019	3.134			.002
Ruggedness				3.597	1,126.0	<.001
D_nest				3.999	31,038.4	<.001
D_neighbor				3.999	6,113.9	<.001
D_powerlines				3.798	101.3	<.001
D_roads				3.980	14,389.5	<.001

Abbreviations: edf, estimated degrees of freedom; *F*, *F* statistics; SE, Standard error; *t*, *t* statistics.

### 3.3 Individual responses to power lines

**FIGURE 3:**
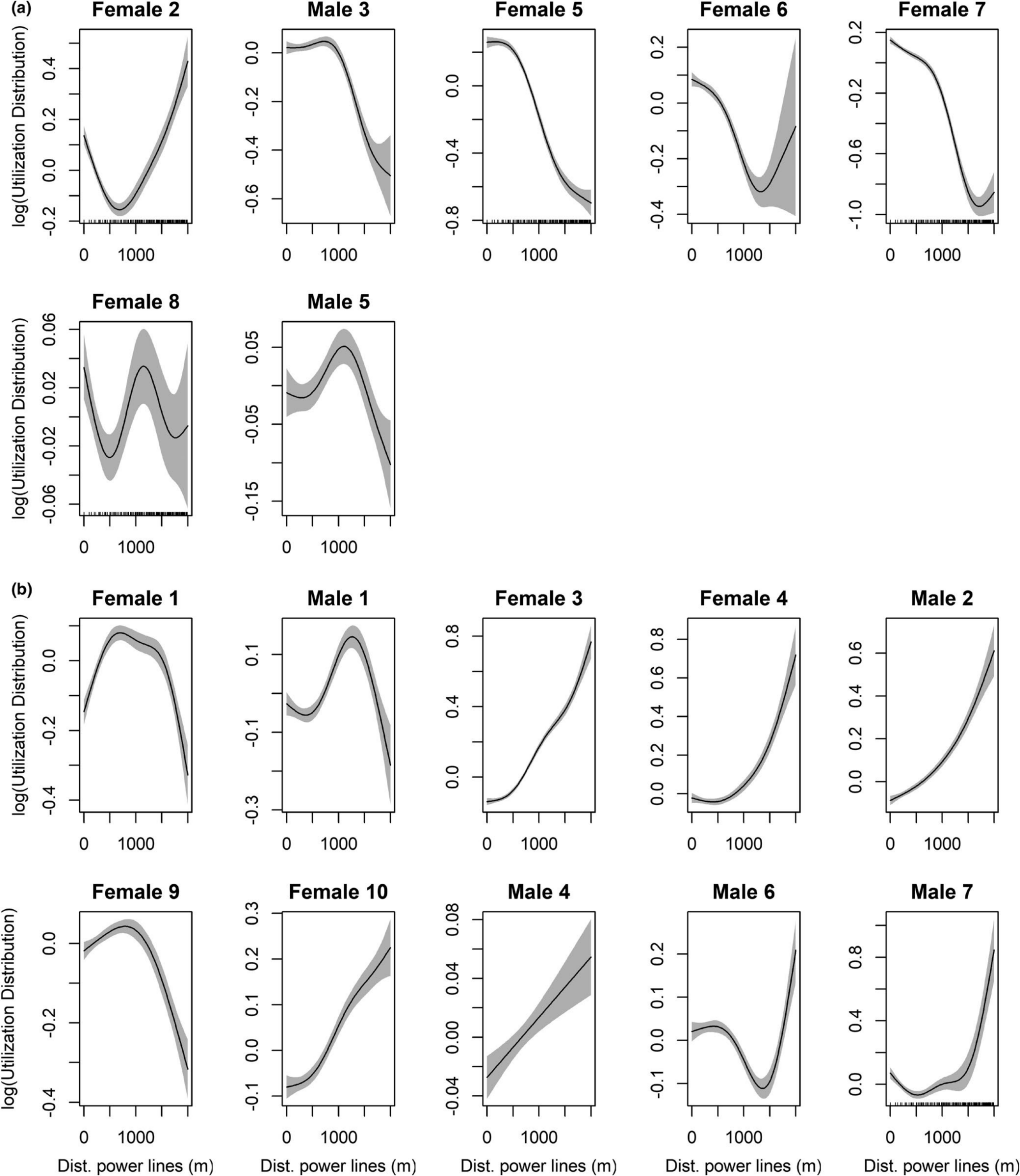
Individual‐level response curves of Bonelli's eagles to power lines inferred from generalized additive models relating the utilization distribution to predictor variables: (a) eagles with increased (attraction) intensity of use, and (b) eagles with decreased (avoidance) of space use near power lines. Distances to power lines considered both the transmission and distribution network. The dataset includes grid cells up to 2 km from power lines. Shaded areas represent 95% confidence intervals

GAMs modeling at the individual level showed patterns broadly similar to the population‐level model, but with important differential responses to the proximity of power lines (*R* ^2^ adjusted (range): 0.453–0.867; Figure 3; Table S3). While seven eagles showed higher intensity of use close to power lines, as observed for the population as a whole, there were other ten for which the intensity of use was lower close to power lines and increased farther away (Figure 3). There was, however, a large variability within each of these two types of behavioral responses. For instance, while the declines in space use close to lines were very marked in females 3, 4, and 10, and in males 2 and 4, the responses were subtler in females 1 and 9, and in males 1, 5, and 7 (Figure 3). Seemingly, the increase in space use close to lines was very marked 5, 6, and 7, and less so for females 2 and 8, and male 6 (Figure 3).

The univariate logistic models (GLM) showed that the probability of an eagle increasing use intensity in the proximity of power lines (attraction) was positively related to the density of transmission lines (*p* = .077; explained deviance = 19.7%) and to the number of transmission pylons (*p* = .092; explained deviance = 16.2%) (Figure S4 and Table S4). The distance of power lines to the nest location, as well as the metrics characterizing the overall power line network and the distribution lines showed no significant effects. There was also no significant difference between males and females in responses to the proximity of power lines (*Q* test, *Q* = 0.615, *p* = .433).

## 4 DISCUSSION

Our study shows that the use of space by adult Bonelli's eagles within their home ranges is strongly affected by the proximity to power lines, after controlling for the effects of other important covariates. However, while at the population level we found a more intensive use of space close to lines, there was marked inter‐individual variability in eagle's responses. In fact, while part of the individuals showed responses consistent with those observed at the population level, others showed reduced intensity of use in the proximity of power lines. Our results also suggest that eagles responding positively to the proximity of power lines had a more extensive network of transmission power lines within their home ranges, but there was a large share of unexplained variation that may be related to individual idiosyncrasies or to unmeasured habitat factors. Overall, these results suggest that inter‐individual variability needs to be duly considered when assessing and mitigating the impacts of power lines and other anthropogenic infrastructures.

### 4.1 Population‐level responses to power lines and other habitat features

At the population level, there was a negative relation between the intensity of space use by Bonelli's eagles and distance to power lines, irrespective of line typology (i.e., distribution or transmission lines). Reasons for this pattern are uncertain, but one possibility is that it was at least partly related to Bonelli's eagles using pylons for hunting and resting, as reported in other birds of prey (Dixon et al., 2013; Infante & Peris, 2003; Slater et al., 2020) and observed in our study area (L. Palma, unpublished data), thereby increasing space use intensity in their vicinity. In fact, these structures provide safe and vantage perching points, as they are largely free from human disturbance and are elevated in relation to the surrounding landscape. Increased use near power lines may also be a consequence of the use of electricity pylons as nesting platforms, as observed in one of our females outside the tracking period, and two other pairs elsewhere in southern Portugal (L. Palma, unpublished data). Alternatively, it is possible that the relation observed did not reflect a direct response to the power lines themselves, but instead was a consequence of unmeasured factors that contribute to attract individuals to their proximity. These may include increased foraging opportunities, as the removal of woody vegetation under and around lines to reduce fire risk may increase habitat suitability for rabbits *Oryctolagus cuniculus* and other prey (Beja et al., 2007; Palma et al., 2013). Overall, while our results suggest that at the level of the whole population there was some attraction of Bonelli's eagles to the proximity of power lines, the identification of the factor, or combination of factors, driving such behavior still needs further investigation.

In contrast to power lines, there was a marked avoidance of roads by Bonelli's eagles, as previously found in studies of nesting habitat selection (Dias et al., 2017), and in studies analyzing species distributions at local and regional scales (López‐López et al., 2006; Muñoz & Real, 2013). This may be a consequence of eagles avoiding human disturbance (Bautista et al., 2004), as roads are regularly used by vehicles and people. Surprisingly, however, the response curve obtained showed a peak in space use at about 2,000 m from roads, declining at longer distances. This pattern is likely an artefact that probably results from the spatial distribution of roads in the study area, but it should be the subject of further research.

The intensity of space use by Bonelli's eagles was positively related to terrain ruggedness, which corresponds to areas with lower human disturbance and where suitable nesting sites are located (Dias et al., 2017; Palma et al., 2013). There was also a positive relation to forested areas, which provide limited foraging resources (Palma et al., 2006), but where Bonelli's eagles in our area find appropriate nesting conditions (Dias et al., 2017; Palma et al., 2013), thereby concentrating a large share of their activity (Bosch et al., 2010; our study). Scrubland was less selected than forests, but still much more used than artificial and agricultural areas, which tend to be avoided by Bonelli's eagles (López‐López et al., 2006; Martínez‐Miranzo et al., 2016). Although scrubland is an important foraging habitat for Bonelli's eagles (Real et al., 2016), where key prey such as rabbits can be hunted (Beja et al., 2007; Palma et al., 2006), it was not selected probably because it represents the dominant land cover (ca. 60%) in our study area. Reasons for the positive, albeit weak selection of waterbodies are unclear, but it is noteworthy that Bonelli's eagles in our study area sometimes hunt gulls (Lariidae), ducks (Anatidae), and other aquatic birds (Palma et al., 2006).

Finally, Bonelli's eagles used more intensively areas close to their nests, as observed in previous studies (Bosch et al., 2010). In contrast, the intensity of use declined close to the nests of conspecifics in neighboring home range, as expected for strongly territorial raptors such as Bonelli's eagles (Bosch et al., 2010; Newton, 2010). Surprisingly, however, the intensity of use peaked at around 6 km from the nearest nest of conspecifics, and declined both at closer and longer distances. This may be because breeding home ranges tend to be aggregated in hilly country with low human population density (e.g., Dias et al., 2017; Palma et al., 2006), and so for pairs breeding at the periphery of the population, at larger distances from conspecific nests, the habitat conditions become less suitable.

### 4.2 Individual responses to power lines

While at the population level we found space use intensity by Bonelli's eagles declining with distance to power lines, analysis at the individual level revealed opposite responses by different individuals. In fact, while part of the eagles tracked increased space use in the proximity to lines, following the population‐level trend, others reduced such use and thus appeared to avoid the proximity of power lines. The occurrence of individuals with such contrasting responses is probably responsible for the wide confidence intervals observed around the mean response curve estimated by the global model (see Figure 2). These confidence intervals were much wider than those estimated for the other covariates, for which there was no appreciable variation in response by different individuals.

To the best of our knowledge, the apparent avoidance of power lines by some Bonelli's eagles had never been demonstrated for birds of prey, although it agrees with previous research showing negative responses to power lines by a few other species (e.g., little bustard *Tetrax tetrax*; Silva et al., 2010, or greater sage‐grouse *Centrocercus urophasianus*; Kohl et al., 2019), and the avoidance of other artificial tall structures such as wind turbines by birds of prey (Marques et al., 2020; Pearce‐Higgins et al., 2009). Our study is also the first describing inter‐individual variability in responses to anthropogenic structures by birds of prey, which is a likely consequence of most habitat selection studies being made at the population level, only reporting average responses across multiple individuals (e.g., Rollan et al., 2010; Tikkanen et al., 2018). However, variations in individual behavior in human‐dominated environments have previously been found in mammalian carnivores (Carricondo‐Sanchez et al., 2020; Gehrt et al., 2010; Sih et al., 2004), as well as in species colonizing urban environments (Lowry et al., 2013; Sol et al., 2013).

Reasons for the inter‐individual differences observed in our study are uncertain, with our results suggesting that they are unrelated to the sex of individuals, while they may be influenced by the characteristics of the power grid within home ranges. Specifically, we found that individuals showing attraction behavior tended to be associated with higher densities of transmission lines and a higher number of transmission pylons (regardless of the density of distribution lines), which may reflect a habituation effect toward anthropogenic structures that have become prominent features within their home range. In contrast, individuals occupying home ranges where transmission lines are scarce may be less tolerant, thereby causing a reaction of fear toward new structures, that is, neophobia (Biasotto & Kindel, 2018; Walters et al., 2014). These results need to be interpreted with care, however, because they are based on relatively small sample sizes, and the models explained only about 15%–20% of the observed variation, which suggest that other important factors may be at play. For instance, the differences observed may be affected to an unknown extent by individual idiosyncrasies (Hertel et al., 2020), as reduced neophobia to anthropogenic objects has been observed in bold, aggressive, and exploratory individuals that are more likely to tolerate anthropogenic disturbances (Merrick & Koprowski, 2017). Although life stage is also known to affect behavioral responses to anthropogenic structures, with higher tolerance by juveniles and dispersing individuals (e.g., Carvalho et al., 2018; Rio‐Maior et al., 2019), this was not an issue in our study because we have only considered breeding adults.

Specific events may also have affected the interaction of individuals with power lines. For instance, a pair started nesting on a transmission pylon in our area after repeated wildfires destroyed the previous nests and the great majority of large trees available in the territory (L. Palma, unpublished data). This pair perched frequently in the electric pylons and nested in a tree below the transmission line before started nesting on an electric pylon, which supports the idea of a progressive habituation to the structure. Other unmeasured differences between home ranges may also have affected the differential responses by individuals, including the spatial patterns of prey availability and foraging areas, the distribution of suitable perches and nest sites, human disturbance, among others. Finally, the patterns observed may be affected to some extent by power lines “avoiding" the eagles, rather than the reverse, because in four of the tracked Bonelli's eagles with reduced space use near power lines, new transmission lines were built following a route that avoided nesting sites, and thus the core of the eagles’ home range. Overall, therefore, although our study strongly supports the presence of individual variation in the Bonelli's eagle responses to power lines, additional research is still needed to understand its causality.

### 4.3 Conservation and management implications

Our results have important implications for evaluating and mitigating impacts of power lines crossing home ranges of Bonelli's eagles and eventually other birds of prey. The study suggests that many individuals are attracted to the proximity of power lines, supporting the importance of deploying raptor‐friendly pylon designs to avoid mortality through electrocution, a major driver of population declines (Chevallier et al., 2015; Hernández‐Matías et al., 2015). Once pylons are safe, they may provide valuable resting and hunting places, and in the case of large pylons they may also provide suitable nesting platforms (L. Palma, unpublished data). Although attraction to power lines may also increase the risk of collision with overhead cables, this has seldom been reported in this species. In any case, under a precautionary approach it may be important to use wire‐marking devices to minimize collision risks, at least in areas where eagles interact more often with power lines (Bernardino et al., 2019; Slater et al., 2020). Our results also suggest that some individuals may show exclusion effects, with reduced space use within about 1 km of power lines, although in some cases this effect may be an artefact resulting from newly built lines avoiding the centers of activity of eagles. Still, apparent exclusion seems to occur mainly in home ranges with few transmission lines, which reinforces that idea that particularly in such cases the routing of new power lines should strongly avoid core activity areas such as around nesting sites.

Our study adds to increasing evidence pointing out the occurrence of significant inter‐individual variation in behavioral traits within a population, which may have profound consequences for wildlife management in human‐dominated landscapes (Merrick & Koprowski, 2017). This should be particularly important in species occurring at low density such as the Bonelli's eagle and other top avian predators, where even impacts to relatively few individuals may affect long‐term population viability (Hernández‐Matías et al., 2015; Sergio et al., 2004). Therefore, when planning new power lines and other infrastructures, it should be recognized that different individuals may respond differently to the same stressors, thereby requiring mitigation strategies that are adjusted to multiple behavioral traits. Although taking such approach is challenging, such individual‐based strategy should contribute to maintain the behavioral heterogeneity that is essential for long‐term population persistence under environmental change (Hertel et al., 2020; Merrick & Koprowski, 2017).

## CONFLICT OF INTEREST

The authors declare that they have no known competing financial interests or personal relationships that could have appeared to influence the work reported in this study.

## AUTHOR CONTRIBUTIONS

**Ana Teresa Marques:** Conceptualization (equal); Data curation (equal); Formal analysis (equal); Investigation (equal); Methodology (equal); Writing – original draft (equal). **Luís Palma:** Data curation (equal); Investigation (equal); Writing – review & editing (equal). **Rui Lourenço:** Data curation (equal); Investigation (equal). **Rogério Cangarato:** Investigation (equal). **Alexandre Leitão:** Investigation (equal). **Miguel Mascarenhas:** Investigation (equal). **João Tiago Tavares:** Investigation (equal). **Ricardo Tomé:** Investigation (equal). **Francisco Moreira:** Conceptualization (equal); Methodology (equal); Supervision (equal); Writing – review & editing (equal). **Pedro Beja:** Conceptualization (equal); Funding acquisition (equal); Methodology (equal); Supervision (equal); Writing – original draft (equal).

## Supporting information

Supplementary Material

## Data Availability

The dataset used in this study is publicly available in Dyrad Digital Repository (https://doi.org/10.5061/dryad.0rxwdbs2x).
