# Supplementary material for: Individual variability in space use near power lines by a long‐lived territorial raptor
Source: Ecol Evol. 2022 Apr 7;12(4):e8811. doi: 10.1002/ece3.8811 (PMC8987490; doi:10.1002/ece3.8811)
Supplement: Supplementary file 1 — Supplementary Material [file ECE3-12-e8811-s001.docx]

Supplementary Information

Individual variability in space use near power lines by a long-lived territorial raptor

Ana Teresa Marques, Luís Palma, Rui Lourenço, Rogério Cangarato, Alexandre Leitão, Miguel Mascarenhas, João Tiago Tavares, Ricardo Tomé, Francisco Moreira, Pedro Beja

*Ecology and Evolution* (2022)

Index

[Table S1 2](#_Toc93315043)

[Table S2 3](#_Toc93315044)

[Table S3 4](#_Toc93315045)

[Table S4 9](#_Toc93315046)

[Fig. S1 10](#_Toc93315059)

[Fig. S2 11](#_Toc93315060)

[Fig. S3 12](#_Toc93315061)

[Fig. S4 21](#_Toc93315062)

**Table S1.** Summary of Bonellis’ eagles tracking data. Minimum distance travelled per day and Utilization Distribution (UD) were computed with the ‘move’ package (Kranstauber and Smolla, 2017). Home range was delimited considering the 95% UD and core area was delimited with the 50% UD. Utilization Distribution: BBMM – Brownian bridge movement models.

| Bird | Tacking period (years) | Tacking period (no. days) | GPS fixes | Minimum distance travelled per day | Home Range (km^2^) | Core Area (km^2^) |
| --- | --- | --- | --- | --- | --- | --- |
| Female 1 | 2009 – 2012 | 1078 | 9603 | 18.0 | 126.0 | 21.5 |
| Female 2 | 2008 – 2012 | 1422 | 12642 | 12.4 | 53.4 | 5.1 |
| Male 1 | 2008 – 2011 | 888 | 9939 | 27.7 | 134.7 | 21.3 |
| Female 3 | 2006 – 2007 | 605 | 2761 | 9.4 | 154.7 | 5.4 |
| Female 4 | 2011 – 2014 | 1146 | 13417 | 21.1 | 116.4 | 4.7 |
| Male 2 | 2007 – 2009 | 703 | 7011 | 25.0 | 165.1 | 20.3 |
| Male 3 | 2011 – 2013 | 467 | 1256 | 4.5 | 128.4 | 15.8 |
| Female 5 | 2010 – 2012 | 736 | 5971 | 14.3 | 100.1 | 4.6 |
| Female 6 | 2010 – 2012 | 738 | 5923 | 15.7 | 112.9 | 10.6 |
| Female 7 | 2008 – 2012 | 1299 | 9019 | 13.6 | 99.8 | 8.2 |
| Female 8 | 2008 – 2010 | 564 | 4623 | 16.9 | 109.7 | 8.6 |
| Female 9 | 2008 – 2012 | 1287 | 12134 | 22.9 | 119.4 | 21.6 |
| Female 10 | 2009 – 2020 | 3826 | 39523 | 19.8 | 146.6 | 9.4 |
| Male 4 | 2008 – 2012 | 1245 | 12657 | 29.6 | 209.9 | 28.9 |
| Male 5 | 2007 – 2010 | 602 | 4175 | 23.2 | 388.3 | 43.7 |
| Male 6 | 2009 – 2012 | 1010 | 7241 | 17.9 | 166.6 | 26.0 |
| Male 7 | 2008 – 2011 | 480 | 4078 | 15.5 | 75.5 | 9.1 |
| Total | 2006 - 2020 | 18096 | 161973 |  |  |  |
| Mean |  | 1064.5 | 9527.8 | 18.1 | 141.6 | 15.6 |
| SD |  | 753.5 | 8315.1 | 6.3 | 71.1 | 10.5 |
| Females | 2006 - 2020 | 12701 | 115616 |  |  |  |
| Mean |  | 1270.1 | 11561.6 | 16.41 | 113.9 | 10.0 |
| SD |  | 901.0 | 9928.4 | 4.0 | 26.4 | 6.1 |
| Males | 2007 - 2013 | 5395 | 46357 |  |  |  |
| Mean |  | 770.7 | 6622.4 | 20.5 | 181.2 | 23.6 |
| SD |  | 268.8 | 3570.0 | 8.0 | 92.8 | 10.2 |

**Table S2.** Description and summary statistics of variables use to model variation in the intensity of home range use by Bonelli’s eagles, at the population (GAMM) and individual (GAM) levels. Mean ± SD and range are provided for continuous variables; proportion per class is presented for the categorical variable.

| **Variable** | **Description** | **Mean ± SD/ proportion** | **Range** |
| --- | --- | --- | --- |
| Habitat | Habitat class (land-cover categories according to DGT, 2007) | Artificial (class 1): 0.85%  Agriculture (class 2): 12.54%  Forests (class 3.1): 23.39%  Scrublands (class 3.2): 62.44%  Waterbodies (classes 4 and 5): 0.78% | - |
| Ruggedness | Terrain ruggedness (30 m spatial resolution) | 15.2 ± 7.6 | 0 – 54.6 |
| D_nest | Distance to the nest (m) | 5713 ± 3506 | 12 – 38626 |
| D_neighbor | Distance to the closest nest from neighbor territories (m) | 7519 **±** 3274 | 15 – 20678 |
| D_powerlines | Distance to transmission or distribution power lines (m) | 601.7 ± 491.9 | 0 – 2000 |
| D_distribution_powerlines | Distance to distribution power lines (m) | 773 ± 673 | 0 – 4342 |
| D_roads | Distance to paved roads (m)  (classes: motorway, trunk, primary, secondary and tertiary, according to Open Street Map; Haklay and Weber, 2008) | 970 ± 865 | 0 – 4478 |

**Table S3.** Summary statistics of Generalized Additive Models (GAM) relating variation in space use intensity of each tracked Bonelli’s eagle to environmental variables (Table S2). SE: Standard error, *t*: *t-*statistics, edf: estimated degrees of freedom, *F*: *F*-statistics, *p*: *p-*value.

| Eagle | Model coefficients | Estimate | SE | *t* | edf | *F* | *p* |
| --- | --- | --- | --- | --- | --- | --- | --- |
| Female 1  *R*^2^ adjusted = 0.453 | Intercept | -9.899 | 0.013 | -746.77 |  |  | <0.001 |
|  | Habitat class (Forest as reference class) | | | | | | |
|  | Artificial | 0.240 | 0.267 | 0.901 |  |  | 0.368 |
|  | Agriculture | -0.225 | 0.024 | -9.461 |  |  | <0.001 |
|  | Scrublands | 0.0170 | 0.015 | 1.111 |  |  | 0.267 |
|  | Ruggedness |  |  |  | 3.891 | 80.2 | <0.001 |
|  | D_nest |  |  |  | 3.996 | 1628.0 | <0.001 |
|  | D_neighbor |  |  |  | 3.992 | 442.8 | <0.001 |
|  | D_powerlines |  |  |  | 3.822 | 41.5 | <0.001 |
|  | D_roads |  |  |  | 3.758 | 79.6 | <0.001 |
| Female 2  *R*^2^ adjusted = 0.834 | Intercept | -9.219 | 0.017 | -532.3 |  |  | <0.001 |
|  | Habitat class (Forest as reference class) | | | | | | |
|  | Artificial | -0.255 | 0.079 | -3.228 |  |  | 0.001 |
|  | Agriculture | -0.209 | 0.030 | -6.910 |  |  | <0.001 |
|  | Scrublands | -0.145 | 0.019 | -7.462 |  |  | <0.001 |
|  | Waterbodies | -1.413 | 0.073 | -19.432 |  |  | 0.025 |
|  | Ruggedness |  |  |  | 1.003 | 10.23 | 0.001 |
|  | D_nest |  |  |  | 3.903 | 3188.18 | <0.001 |
|  | D_neighbor |  |  |  | 3.945 | 573.98 | <0.001 |
|  | D_powerlines |  |  |  | 3.737 | 71.80 | <0.001 |
|  | D_roads |  |  |  | 3.940 | 122.08 | <0.001 |
| Male 1  R^2^ adjusted = 0.577 | Intercept | -10.173 | 0.017 | -588.263 |  |  | <0.001 |
|  | Habitat class (Forest as reference class) | | | | | | |
|  | Artificial | -0.225 | 0.100 | -2.249 |  |  | <0.001 |
|  | Agriculture | -0.014 | 0.028 | -0.506 |  |  | 0.025 |
|  | Scrublands | 0.137 | 0.019 | 7.331 |  |  | 0.613 |
|  | Waterbodies | 0.0234 | 0.045 | 5.185 |  |  | <0.001 |
|  | Ruggedness |  |  |  | 3.765 | 219.39 | <0.001 |
|  | D_nest |  |  |  | 3.981 | 1488.99 | <0.001 |
|  | D_neighbor |  |  |  | 3.995 | 357.29 | <0.001 |
|  | D_powerlines |  |  |  | 3.806 | 32.86 | <0.001 |
|  | D_roads |  |  |  | 3.691 | 302.56 | <0.001 |
| Female 3  *R*^2^ adjusted = 0.806 | Intercept | -10.562 | 0.006 | -163.416 |  |  | <0.001 |
|  | Habitat class (Forest as reference class) | | | | | | |
|  | Artificial | -0.418 | 0.032 | -13.233 |  |  | <0.001 |
|  | Agriculture | -0.390 | 0.014 | -28.193 |  |  | <0.001 |
|  | Scrublands | -0.214 | 0.009 | -23.001 |  |  | <0.001 |
|  | Waterbodies | -0.824 | 0.095 | -8.714 |  |  | <0.001 |
|  | Ruggedness |  |  |  | 3.888 | 156.3 | <0.001 |
|  | D_nest |  |  |  | 3.997 | 6918.8 | <0.001 |
|  | D_neighbor |  |  |  | 3.995 | 965.2 | <0.001 |
|  | D_powerlines |  |  |  | 3.815 | 389.3 | <0.001 |
|  | D_roads |  |  |  | 2.985 | 502.2 | <0.001 |
| Female 4  *R*^2^ adjusted = 0.735 | Intercept | -10.190 | 0.009 | -1078.357 |  |  | <0.001 |
|  | Habitat class (Forest as reference class) | | | | | | |
|  | Artificial | -0.198 | 0.045 | -4.449 |  |  | <0.001 |
|  | Agriculture | -0.281 | 0.017 | -16.685 |  |  | <0.001 |
|  | Scrublands | -0.353 | 0.013 | -26.631 |  |  | <0.001 |
|  | Waterbodies | -0.502 | 0.082 | -6.089 |  |  | <0.001 |
|  | Ruggedness |  |  |  | 3.898 | 284.40 | <0.001 |
|  | D_nest |  |  |  | 3.998 | 3218.36 | <0.001 |
|  | D_neighbor |  |  |  | 3.947 | 515.44 | <0.001 |
|  | D_powerlines |  |  |  | 3.562 | 54.55 | <0.001 |
|  | D_roads |  |  |  | 3.925 | 63.95 | <0.001 |
| Male 2  R^2^ adjusted = 0.764 | Intercept | -10.317 | 0.008 | -127.344 |  |  | <0.001 |
|  | Habitat class (Forest as reference class) | | | | | | |
|  | Artificial | -0.490 | 0.034 | -14.333 |  |  | <0.001 |
|  | Agriculture | -0.349 | 0.015 | -23.949 |  |  | <0.001 |
|  | Scrublands | -0.320 | 0.011 | -28.498 |  |  | <0.001 |
|  | Waterbodies | -0.596 | 0.089 | -6.703 |  |  | <0.001 |
|  | Ruggedness |  |  |  | 3.920 | 295.74 | <0.001 |
|  | D_nest |  |  |  | 3.988 | 6089.93 | <0.001 |
|  | D_neighbor |  |  |  | 3.992 | 1896.81 | <0.001 |
|  | D_powerlines |  |  |  | 3.229 | 106.22 | <0.001 |
|  | D_roads |  |  |  | 3.834 | 85.16 | <0.001 |
| Male 3  *R*^2^ adjusted = 0.649 | Intercept | -9.984 | 0.011 | -932.895 |  |  | <0.001 |
|  | Habitat class (Forest as reference class) | | | | | | |
|  | Artificial | -0.197 | 0.048 | -4.105 |  |  | <0.001 |
|  | Agriculture | -0.149 | 0.017 | -8.601 |  |  | <0.001 |
|  | Scrublands | -0.240 | 0.014 | -16.713 |  |  | <0.001 |
|  | Waterbodies | -0.331 | 0.080 | -4.143 |  |  | <0.001 |
|  | Ruggedness |  |  |  | 3.783 | 260.96 | <0.001 |
|  | D_nest |  |  |  | 3.998 | 2094.25 | <0.001 |
|  | D_neighbor |  |  |  | 3.959 | 640.08 | <0.001 |
|  | D_powerlines |  |  |  | 3.736 | 57.64 | <0.001 |
|  | D_roads |  |  |  | 3.949 | 49.91 | <0.001 |
|  |  |  |  |  |  |  |  |
|  |  |  |  |  |  |  |  |
|  |  |  |  |  |  |  |  |
|  |  |  |  |  |  |  |  |
| Female 5  *R*^2^ adjusted = 0.751 | Intercept | -10.559 | 0.017 | -618.95 |  |  | <0.001 |
|  | Habitat class (Forest as reference class) | | | | | | |
|  | Artificial | 0.648 | 0.082 | 7.941 |  |  | <0.001 |
|  | Agriculture | 0.393 | 0.027 | 14.413 |  |  | <0.001 |
|  | Scrublands | 0.988 | 0.019 | 21.213 |  |  | <0.001 |
|  | Waterbodies | 0.712 | 0.078 | 9.083 |  |  | <0.001 |
|  | Ruggedness |  |  |  | 3.798 | 125.5 | <0.001 |
|  | D_nest |  |  |  | 3.986 | 3618.8 | <0.001 |
|  | D_neighbor |  |  |  | 3.963 | 195.1 | <0.001 |
|  | D_powerlines |  |  |  | 3.875 | 321.3 | <0.001 |
|  | D_roads |  |  |  | 3.731 | 245.6 | <0.001 |
| Female 6  *R*^2^ adjusted = 0.622 | Intercept | -10.113 | 0.012 | -819.260 |  |  | <0.001 |
|  | Habitat class (Forest as reference class) | | | | | | |
|  | Artificial | -0.420 | 0.047 | -9.012 |  |  | <0.001 |
|  | Agriculture | -0.055 | 0.021 | -2.666 |  |  | 0.008 |
|  | Scrublands | 0.114 | 0.015 | 7.622 |  |  | <0.001 |
|  | Waterbodies | 0.127 | 0.076 | 1.664 |  |  | 0.096 |
|  | Ruggedness |  |  |  | 3.356 | 6.506 | <0.001 |
|  | D_nest |  |  |  | 3.997 | 2171.523 | <0.001 |
|  | D_neighbor |  |  |  | 3.879 | 263.23832 | <0.001 |
|  | D_powerlines |  |  |  | 3.630 | 81.423 | <0.001 |
|  | D_roads |  |  |  | 1.003 | 2659.223 | <0.001 |
| Female 7  *R*^2^ adjusted = 0.846 | Intercept | -10.315 | 0.0175 | -589.763 |  |  | <0.001 |
|  | Habitat class (Forest as reference class) | | | | | | |
|  | Artificial | -0.153 | 0.049 | -3.163 |  |  | 0.002 |
|  | Agriculture | -0.119 | 0.024 | -4.976 |  |  | <0.001 |
|  | Scrublands | 0.121 | 0.019 | 6.442 |  |  | <0.001 |
|  | Waterbodies | -0.224 | 0.032 | -7.020 |  |  | <0.001 |
|  | Ruggedness |  |  |  | 3.160 | 13.66 | <0.001 |
|  | D_nest |  |  |  | 3.997 | 7132.31 | <0.001 |
|  | D_neighbor |  |  |  | 3.994 | 5933.14 | <0.001 |
|  | D_powerlines |  |  |  | 0.937 | 291.27 | <0.001 |
|  | D_roads |  |  |  | 3.923 | 118.46 | <0.001 |
| Female 8  R^2^ adjusted = 0.867 | Intercept | -10.056 | 0.016 | -610.118 |  |  | <0.001 |
|  | Habitat class (Forest as reference class) | | | | | | |
|  | Artificial | -0.224 | 0.038 | -5.816 |  |  | <0.001 |
|  | Agriculture | -0.359 | 0.021 | -17.134 |  |  | <0.001 |
|  | Scrublands | -0.268 | 0.018 | -15.119 |  |  | <0.001 |
|  | Waterbodies | -0.201 | 0.027 | -7.329 |  |  | <0.001 |
|  | Ruggedness |  |  |  | 1.000 | 47.916 | <0.001 |
|  | D_nest |  |  |  | 3.941 | 5649.114 | <0.001 |
|  | D_neighbor |  |  |  | 3.994 | 3640.181 | <0.001 |
|  | D_powerlines |  |  |  | 3.649 | 5.079 | <0.001 |
|  | D_roads |  |  |  | 3.906 | 800.397 | <0.001 |
| Female 9  R^2^ adjusted = 0.76 | Intercept | -9.850 | 0.009 | -1045.902 |  |  | <0.001 |
|  | Habitat class (Forest as reference class) | | | | | | |
|  | Artificial | -0.292 | 0.052 | -5.573 |  |  | <0.001 |
|  | Agriculture | 0.057 | 0.022 | 2.659 |  |  | 0.008 |
|  | Scrublands | -0.051 | 0.012 | -4.455 |  |  | <0.001 |
|  | Waterbodies | 0.232 | 0.058 | 3.976 |  |  | <0.001 |
|  | Ruggedness |  |  |  | 3.905 | 46.49 | <0.001 |
|  | D_nest |  |  |  | 3.968 | 5684.23 | <0.001 |
|  | D_neighbor |  |  |  | 3.996 | 2509.05 | <0.001 |
|  | D_powerlines |  |  |  | 3.437 | 32.23 | <0.001 |
|  | D_roads |  |  |  | 3.733 | 285.16 | <0.001 |
| Female 10  *R*^2^ adjusted = 0.597 | Intercept | -10.556 | 0.012 | -855.140 |  |  | <0.001 |
|  | Habitat class (Forest as reference class) | | | | | | |
|  | Artificial | 0.035 | 0.071 | 0.492 |  |  | 0.623 |
|  | Agriculture | -0.041 | 0.019 | -2.145 |  |  | 0.032 |
|  | Scrublands | -0.018 | 0.014 | -1.311 |  |  | 0.190 |
|  | Waterbodies | -0.407 | 0.178 | -2.281 |  |  | 0.023 |
|  | Ruggedness |  |  |  | 2.116 | 339.2 | <0.001 |
|  | D_nest |  |  |  | 3.931 | 2216.5 | <0.001 |
|  | D_neighbor |  |  |  | 3.981 | 2048.8 | <0.001 |
|  | D_powerlines |  |  |  | 3.186 | 52.4 | <0.001 |
|  | D_roads |  |  |  | 3.800 | 137.2 | <0.001 |
| Male 4  *R*^2^ adjusted = 0.723 | Intercept | -10.758 | 0.010 | -104.352 |  |  | <0.001 |
|  | Habitat class (Forest as reference class) | | | | | | |
|  | Artificial | 0.148 | 0.046 | 3.263 |  |  | <0.001 |
|  | Agriculture | 0.058 | 0.015 | 3.949 |  |  | <0.001 |
|  | Scrublands | 0.053 | 0.011 | 4.864 |  |  | <0.001 |
|  | Waterbodies | -0.589 | 0.067 | -8.797 |  |  | <0.001 |
|  | Ruggedness |  |  |  | 1.871 | 224.07 | <0.001 |
|  | D_nest |  |  |  | 3.950 | 6016.48 | <0.001 |
|  | D_neighbor |  |  |  | 3.995 | 5605.31 | <0.001 |
|  | D_powerlines |  |  |  | 1.001 | 19.48 | <0.001 |
|  | D_roads |  |  |  | 3.937 | 169.49 | <0.001 |
| Male 5  *R*^2^ adjusted = 0.566 | Intercept | -11.451 | 0.009 | -1277.980 |  |  | <0.001 |
|  | Habitat class (Forest as reference class) | | | | | | |
|  | Artificial | 0.062 | 0.066 | 0.933 |  |  | 0.351 |
|  | Agriculture | -0.047 | 0.022 | -2.160 |  |  | 0.031 |
|  | Scrublands | 0.1178 | 0.011 | 10.997 |  |  | <0.001 |
|  | Waterbodies | 0.1977 | 0.086 | 2.296 |  |  | 0.022 |
|  | Ruggedness |  |  |  | 3.712 | 386.11 | <0.001 |
|  | D_nest |  |  |  | 3.999 | 8263.59 | <0.001 |
|  | D_neighbor |  |  |  | 3.995 | 3196.28 | <0.001 |
|  | D_powerlines |  |  |  | 3.892 | 36.16 | <0.001 |
|  | D_roads |  |  |  | 3.926 | 113.96 | <0.001 |
| Male 6  *R*^2^ adjusted = 0.664 | Intercept | -10.316 | 0.011 | -981.249 |  |  | <0.001 |
|  | Habitat class (Forest as reference class) | | | | | |  |
|  | Artificial | 0.045 | 0.079 | 0.571 |  |  | 0.568 |
|  | Agriculture | -0.096 | 0.021 | -4.551 |  |  | <0.001 |
|  | Scrublands | 0.068 | 0.013 | 5.412 |  |  | <0.001 |
|  | Waterbodies | -0.120 | 0.112 | -1.079 |  |  | 0.281 |
|  | Ruggedness |  |  |  | 3.923 | 5370858 | <0.001 |
|  | D_nest |  |  |  | 3.993 | 3140.874 | <0.001 |
|  | D_neighbor |  |  |  | 3.982 | 1341.178 | <0.001 |
|  | D_powerlines |  |  |  | 3.496 | 9.526 | <0.001 |
|  | D_roads |  |  |  | 3.741 | 220.973 | <0.001 |
| Male 6  *R*^2^ adjusted = 0.72 | Intercept | -9.788 | 0.019 | -514.338 |  |  | <0.001 |
|  | Habitat class (Forest as reference class) | | | | | | |
|  | Artificial | 0.021 | 0.109 | 0.194 |  |  | 0.846 |
|  | Agriculture | 0.218 | 0.026 | 8.386 |  |  | <0.001 |
|  | Scrublands | 0.154 | 0.021 | 7.250 |  |  | <0.001 |
|  | Waterbodies | 0.160 | 0.064 | 2.513 |  |  | 0.012 |
|  | Ruggedness |  |  |  | 3.117 | 12.45 | <0.001 |
|  | D_nest |  |  |  | 3.994 | 2534.80 | <0.001 |
|  | D_neighbor |  |  |  | 3.986 | 1694.71 | <0.001 |
|  | D_powerlines |  |  |  | 3.860 | 27.40 | <0.001 |
|  | D_roads |  |  |  | 3.899 | 93.50 | <0.001 |

**Table S4.** Summary statistics of univariate Generalised Linear Models (GLM) relating the probability of an eagle having increased space use near power lines (attraction) to variables describing the power grid within home ranges.

| **Model coefficients** | **Estimate** | **SE** | **Z value** | **P value** |
| --- | --- | --- | --- | --- |
| Model 1: Minimum distance between power lines and nest | | | | |
| Intercept | 1.093 | 1.173 | 0.932 | 0.351 |
| D_PowerLines_nest | -0.001 | 0.001 | -1.081 | 0.280 |
| Model 2: Minimum distance between transmission lines and nest | | | | |
| Intercept | 0.457 | 0.676 | 0.675 | 0.500 |
| D_Transmission_nest | -5.677e-05 | 4.822e-05 | -1.177 | 0.239 |
| Model 3: Minimum distance between distribution lines and nest | | | | |
| Intercept | 0.759 | 1.085 | 0.700 | 0.484 |
| D_Distribution_nest | -0.0005 | 0.0005 | -0.883 | 0.377 |
| Model 4: Density of power lines in the home range | | | | |
| Intercept | -0.330 | 1.541 | -0.214 | 0.830 |
| Density_PowerLines | 3.156 | 21.715 | 0.145 | 0.884 |
| Model 5: Density of transmission lines in the home range | | | | |
| Intercept | -1.539 | 0.949 | -1.623 | 0.105 |
| **Density_Transmission** | **119.571** | **67.498** | **1.771** | **0.077** |
| Model 6: Density of distribution lines in the home range | | | | |
| Intercept | -1.306 | 1.736 | -0.752 | 0.452 |
| Density_Distribution | 21.599 | 30.403 | 0.710 | 0.477 |
| Model 7: No. of power line pylons in the home range | | | | |
| Intercept | -0.489 | 1.266 | -0.386 | 0.699 |
| No_pylons | 0.0007 | 0.002 | 0.318 | 0.751 |
| Model 8: No. of transmission pylons in the home range | | | | |
| Intercept | -1.479 | 0.972 | -1.522 | 0.128 |
| **No_Transmission_pylons** | **0.037** | **0.022** | **1.685** | **0.092** |
| Model 9: No. of distribution pylons in the home range | | | | |
| Intercept | -0.185 | 1.234 | -0.150 | 0.881 |
| No_Distribution_pylons | 0.0001 | 0.002 | 0.059 | 0.953 |


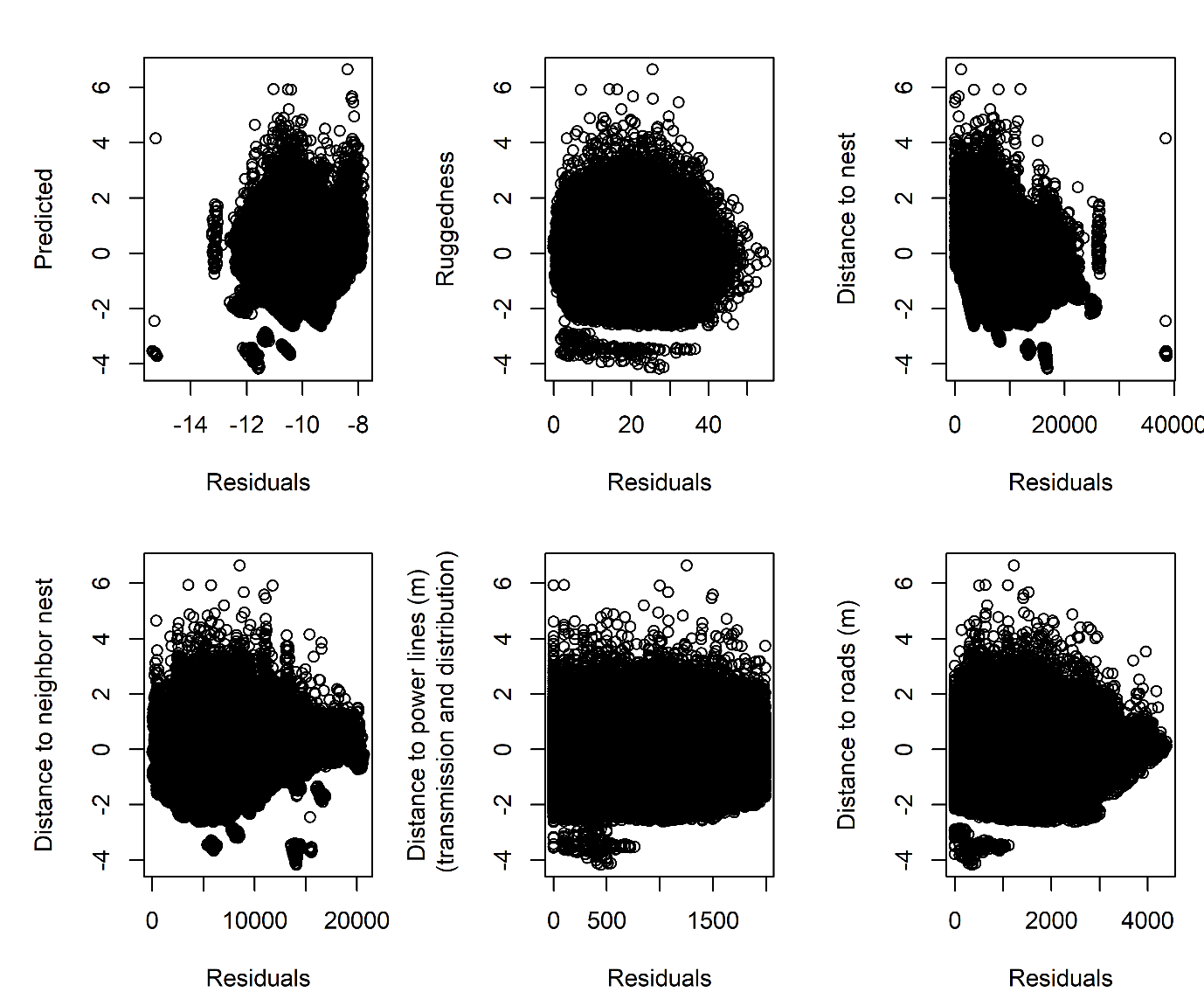


**Fig. S1.** Validation plots for the Generalized additive mixed model partial effects of the predictor variables used to model Bonelli’s eagle Utilization Distribution, using the global power line network (transmission and distribution) (model presented in Fig. 2): residuals versus fitted values and residuals versus explanatory variables.


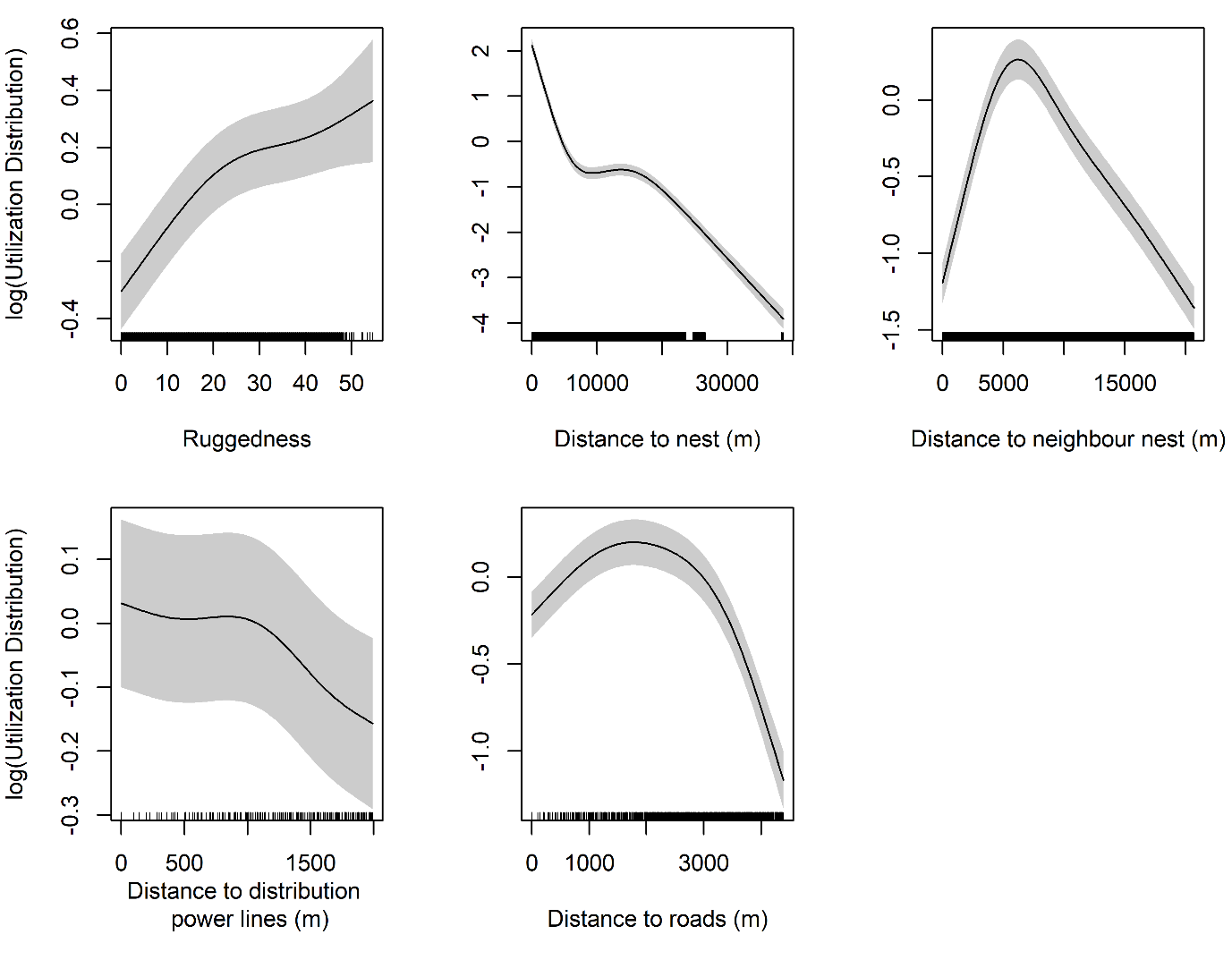


**Fig. S2**. Population-level partial response curves of Bonelli’s eagles inferred from a Generalized Additive Mixed Model relating the Utilization Distribution to predictor variables. Distances to power lines considered only the distribution network. Shaded areas represent 95% confidence intervals. Ticks on the x-axis represent the location of observations along the predictor. *R*^2^ adjusted = 0.486.


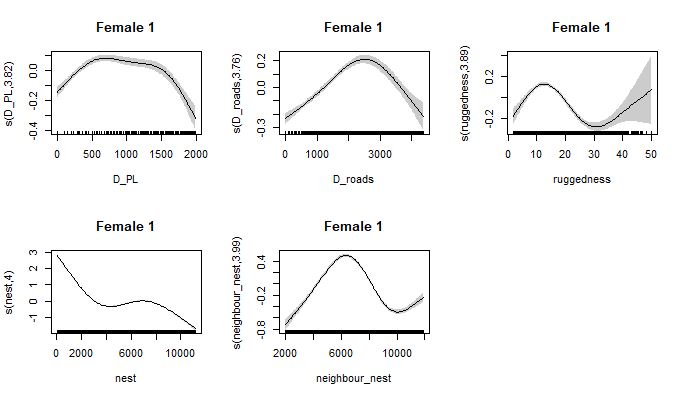


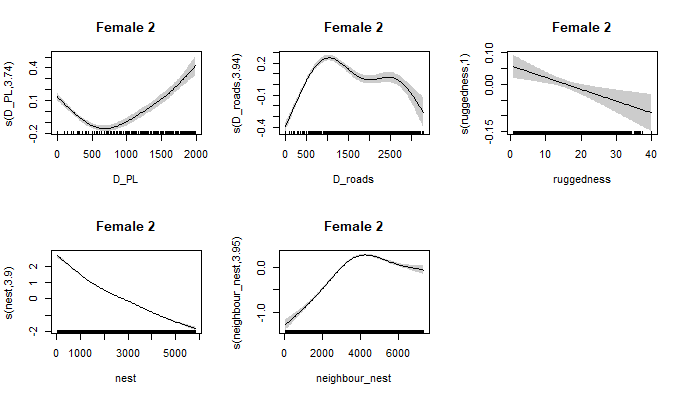


**Fig. S3.** Individual-level partial curves of Bonelli’s eagles inferred from a Generalized Additive Mixed Model relating the Utilization Distribution to predictor variables. Distances to power lines considered both the transmission and the distribution network. Shaded areas represent 95% confidence intervals. Ticks on the x-axis represent the location of observations along the predictor. (cont.)

Fig. S3 (cont.)


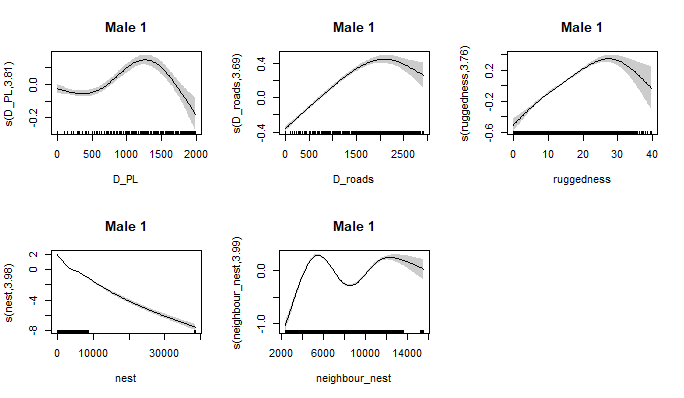


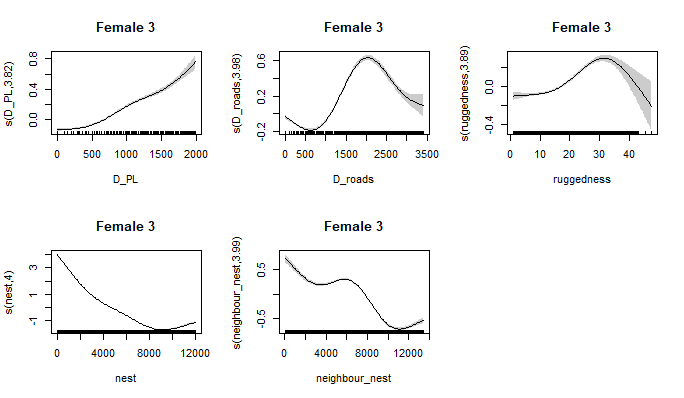


Fig. S3 (cont.)


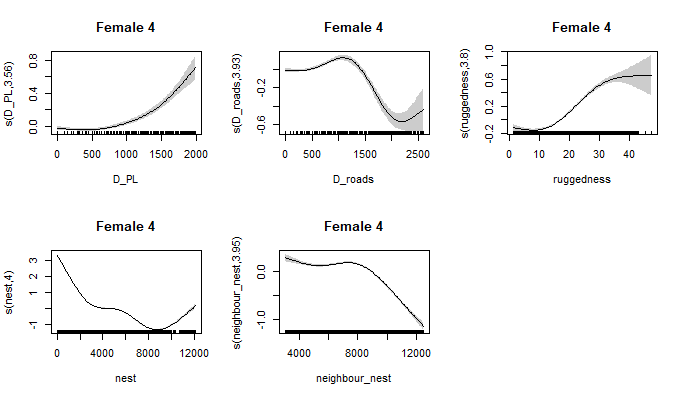

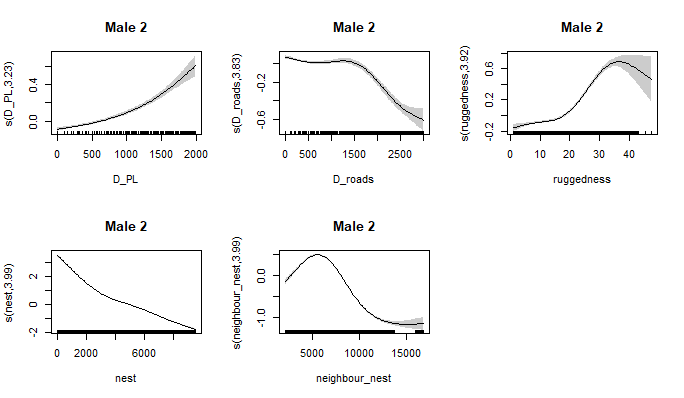


Fig. S3 (cont.)


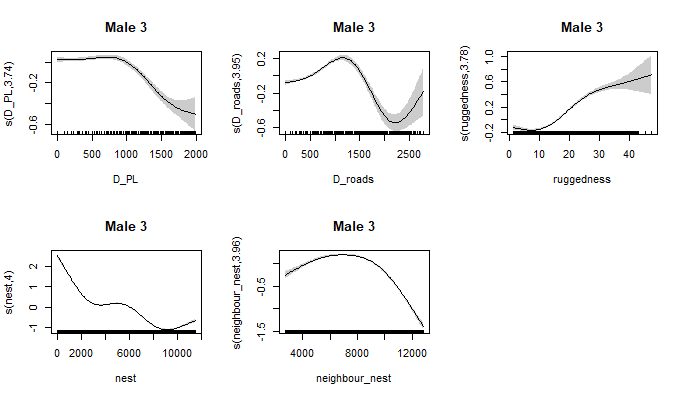


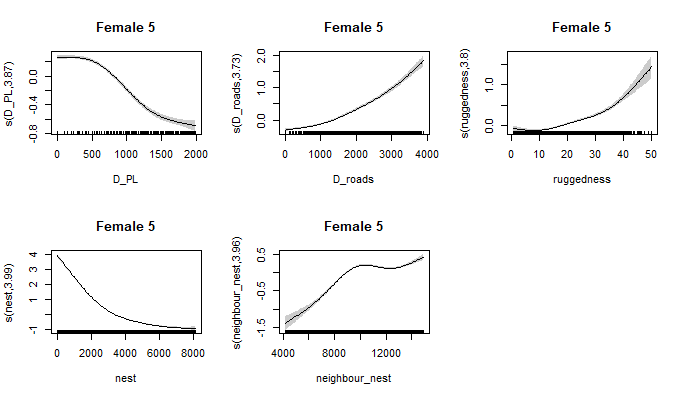


Fig. S3 (cont.)


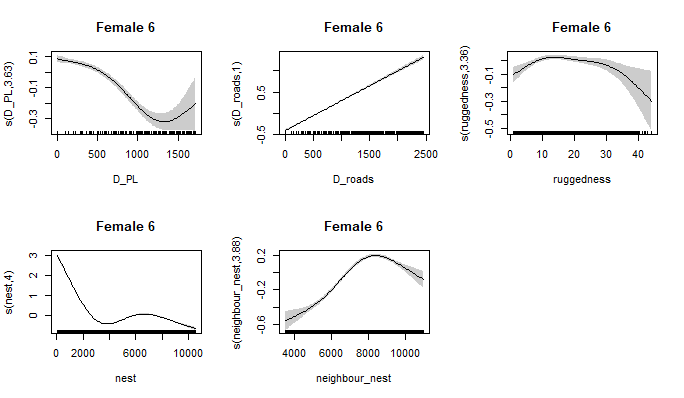

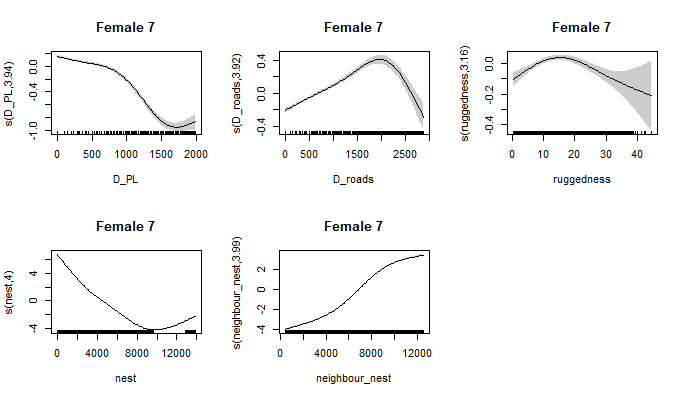


Fig. S3 (cont.)


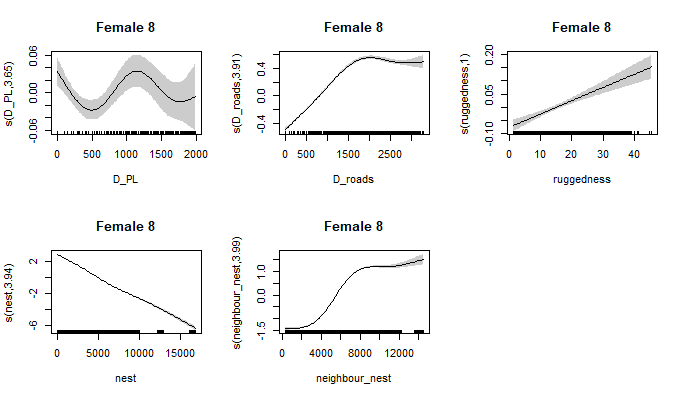

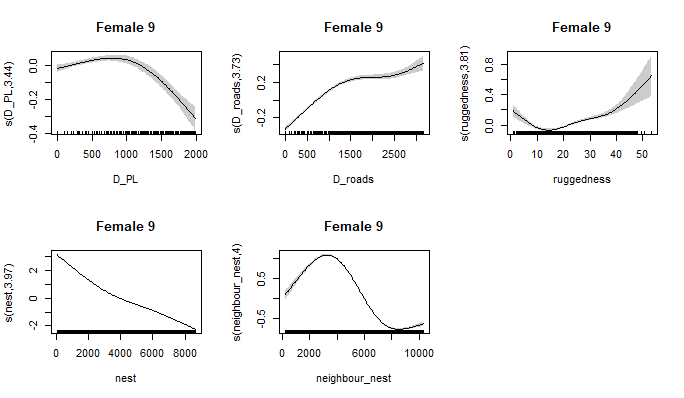


Fig. S3 (cont.)


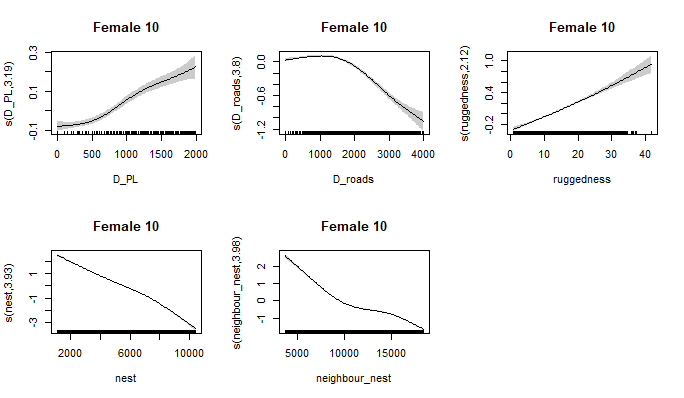


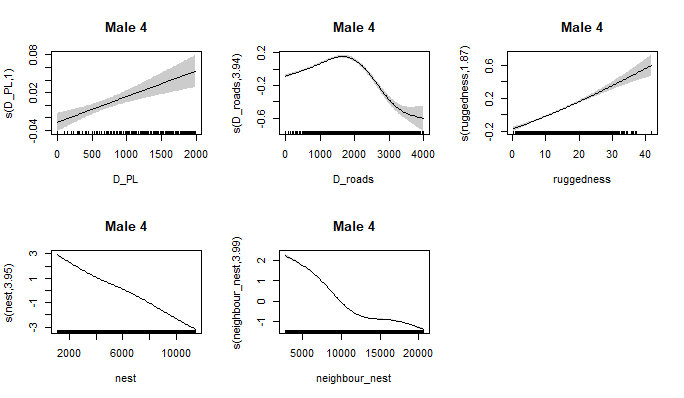


Fig. S3 (cont.)


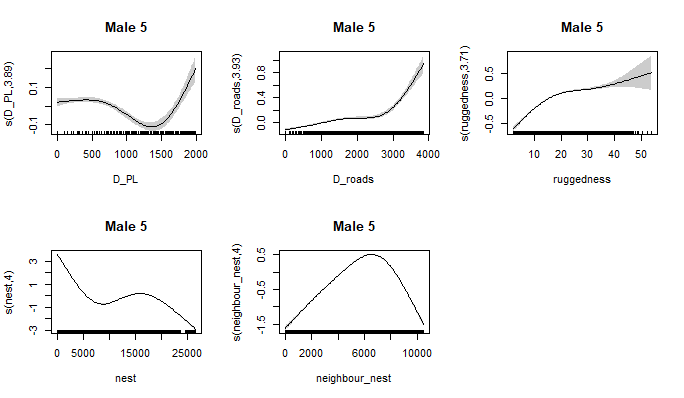

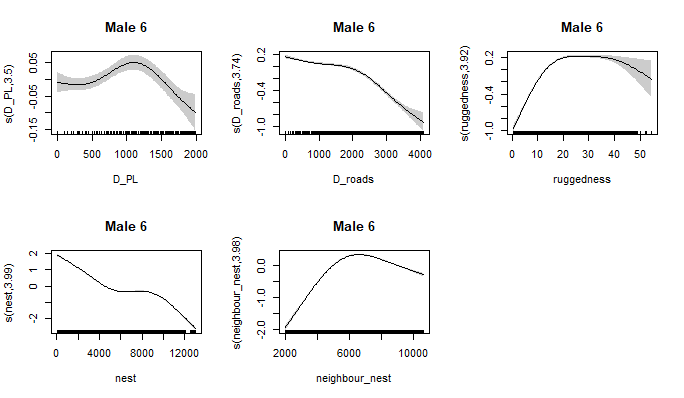


Fig. S3 (cont.)


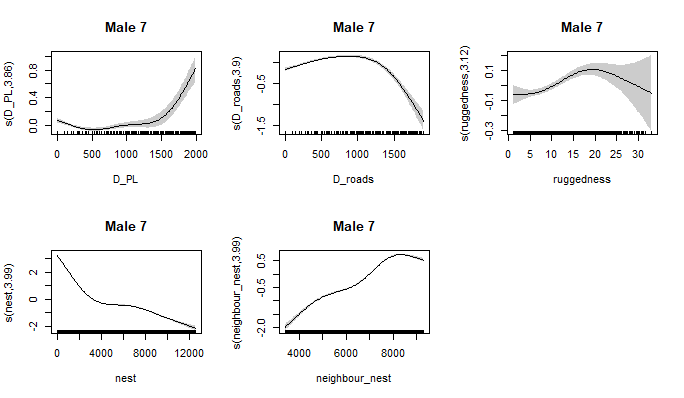


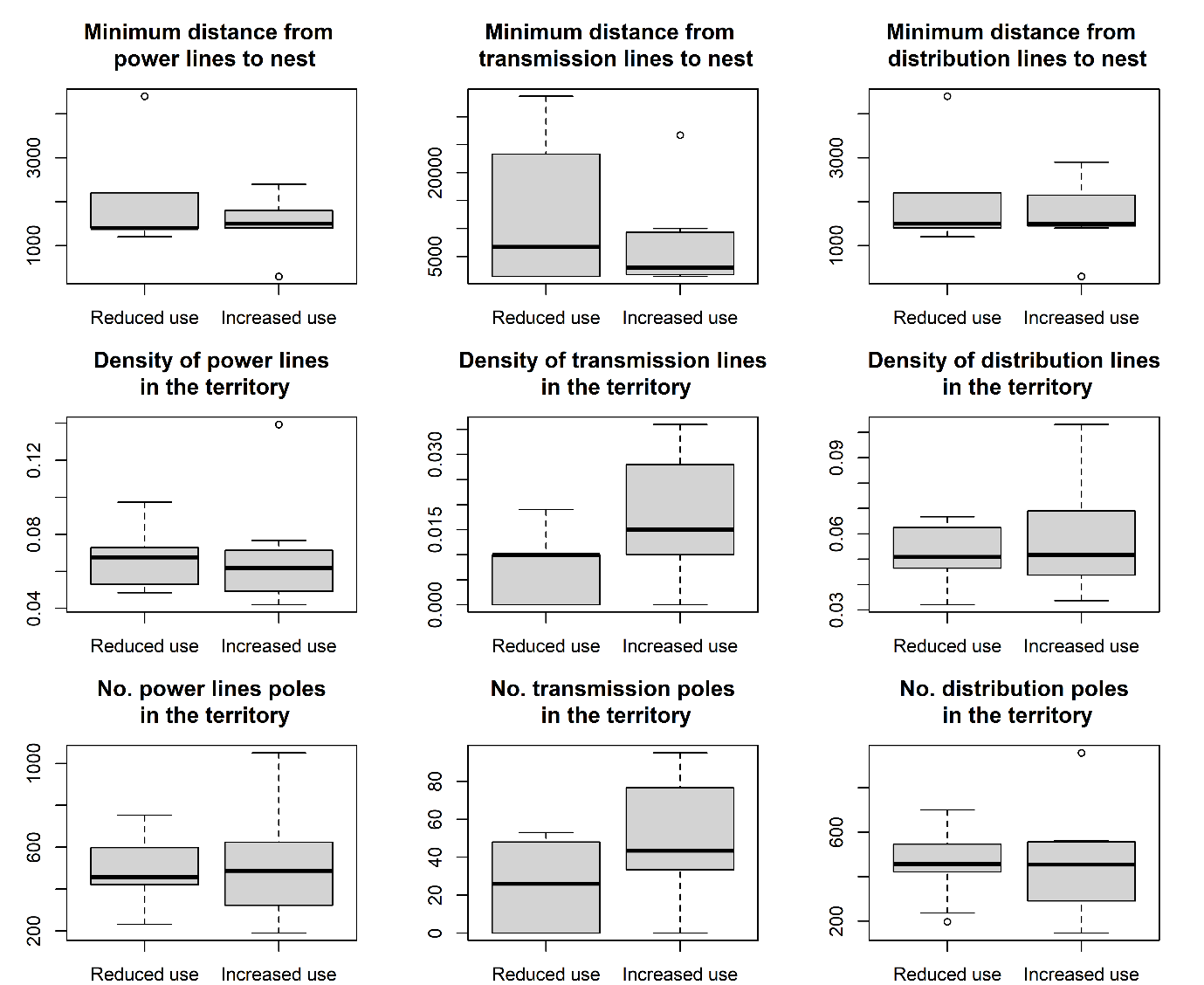


**Fig. S4**. Comparison of power lines characteristics for the home ranges of eagles with increased (attraction) or decreased (avoidance) intensity of space use in the proximity of power lines: minimum distance between power lines and nest, power line density, number of pylons in the home range for (i) all power lines (graphs in the left), (ii) transmission lines (graphs in the center) and (iii) distribution lines (graphs in the right).
